# Supplementary material for: DNA Ligase C and Prim-PolC participate in base excision repair in mycobacteria
Source: Nat Commun. 2017 Nov 1;8:1251. doi: 10.1038/s41467-017-01365-y (PMC5663958; doi:10.1038/s41467-017-01365-y)

Supplementary Table 1. List of DNA primers used in this study.

Primers used for preparation of protein expression constructs

Amplicon	Forward primer 5'->3'	Reverse primer 5'->3'
**Nth**	GGAATTCCATATGAGTGCGGGTGCCGCCCGG	GCCCAAGCTTCACAAACCGGCCAGCGCCA
**FPG (SLIC)**	TTAACTTTAAGAAGGAGATATACCATGCCTGAGCTTCCCGAGGT	TGGTGGTGCTCGAGTGCGGCCGCAAGCTGACCCGAGGCACCCGCGGAC
**MPG**	GGAATTCCATATGAGCGTCGACCTGCTGAC	CGCGGATCCTCAGTCACTGCTGCCGGGCGC
**ExoIII**	GGAATTCCATATGGCTTCCCCGCAGACTCT	CGCGGATCCTCATGCGCTGAACTCGACCG
**XthA**	GGAATTCCATATGCGTGTGGCCACCTGGAA	CGCGGATCCTCAGTTCAGTTCGACGAGCACG
**EndoIV**	GGAATTCCATATGCTCATTGGCTCGCATGTAG	CGCGGATCCCTAACCCGCGTGTTCGCGCA
**XseB**	GGAATTCCATATGAAGCCCATTAGTGAACTGG	GCCGCTCGAGGTCCTCGTCGGCCTCGCGG
**PolA**	GGAATTCCATATGAGCCCCGCCAAGACCGC	GCCCAAGCTTCAGTGAGCCGCGGCGTCCCA
**PolD1**	GGAATTCCATATGACAACCATCGCAGTTGC	CGCGGCCGCTCAGCGGCGCCACTGTGCGCCCG
**Prim-PolC**	GGAATTCCATATGTGATGGCCAGTGCGGCAACC	GCCCAAGCTTTCACTGCTTGCGGTTGCCCTGCT
**LigC1**	GGAATTCCATATGGACTTGCCGGTGCAGCC	GCCCAAGCTTTCACTGTTCCTCCAGCACGTCGT
**Prim-PolC GFP (SLIC)**	GGAGGATCCGAACGATGGCCAGTGCGGCAACCGAA	CCTTAAGCTTCTGCTTGCGGTTGCCCTGCT
**LigC1 GFP (SLIC)**	TCGCTACTCTCATCGTGGAATCCTGACAGGATCGCCAGGGAGGATCCGAACGATGGACTTGCCGGTGCAGCC	TGTACAGCGAGGTGATGTCGGCGGCCTTAAGCTTCTGTTCCTCCAGCACGTCGT

Primers used for generation of gene replacement plasmids

Amplicon	Forward primer 5'->3'	Reverse primer 5'->3'
**ΔPolD2 (MSMEG_0597) LF**	AACTGCAGCGACATCGCCGGACGACATC	CAAGCTTGAGGATCATCGGCCTGCCG
**RF**	GCTGATCGAGATCGCCCGC	GAAGATCTAGGCGCCATCACGGTCGG
**ΔPrim-PolC (MSMEG_6301) LF**	TGGAGACCCGCACCCTGATG	GGCGAGCGGTTGTTCATCG
**RF**	GTCCGGGTTGGTGAACCGG	TCATACGACATCCCCGGCGG
**ΔLigC2 (MSMEG_6304) LF**	AGACCAGACAGCCCGAGATCGC	TCATACGACATCCCCGGCGG
**RF**	CGACGTTCCACCTGACGCC	CGTGGGCCTGACCGATCAG
**ΔLigC1 (MSMEG_6302),LigC2 LF**	AGACCAGACAGCCCGAGATCGC	CGAGGCCGTCGACAACAAACG
**RF**	CGACGTTCCACCTGACGCC	CGTGGGCCTGACCGATCAG
**ΔLigC1,LigC2,Prim-PolC LF**	TGGAGACCCGCACCCTGATG	GGCGAGCGGTTGTTCATCG
**RF**	CGATCTGCTGGCACTCGGC	CGTGGGCCTGACCGATCAG

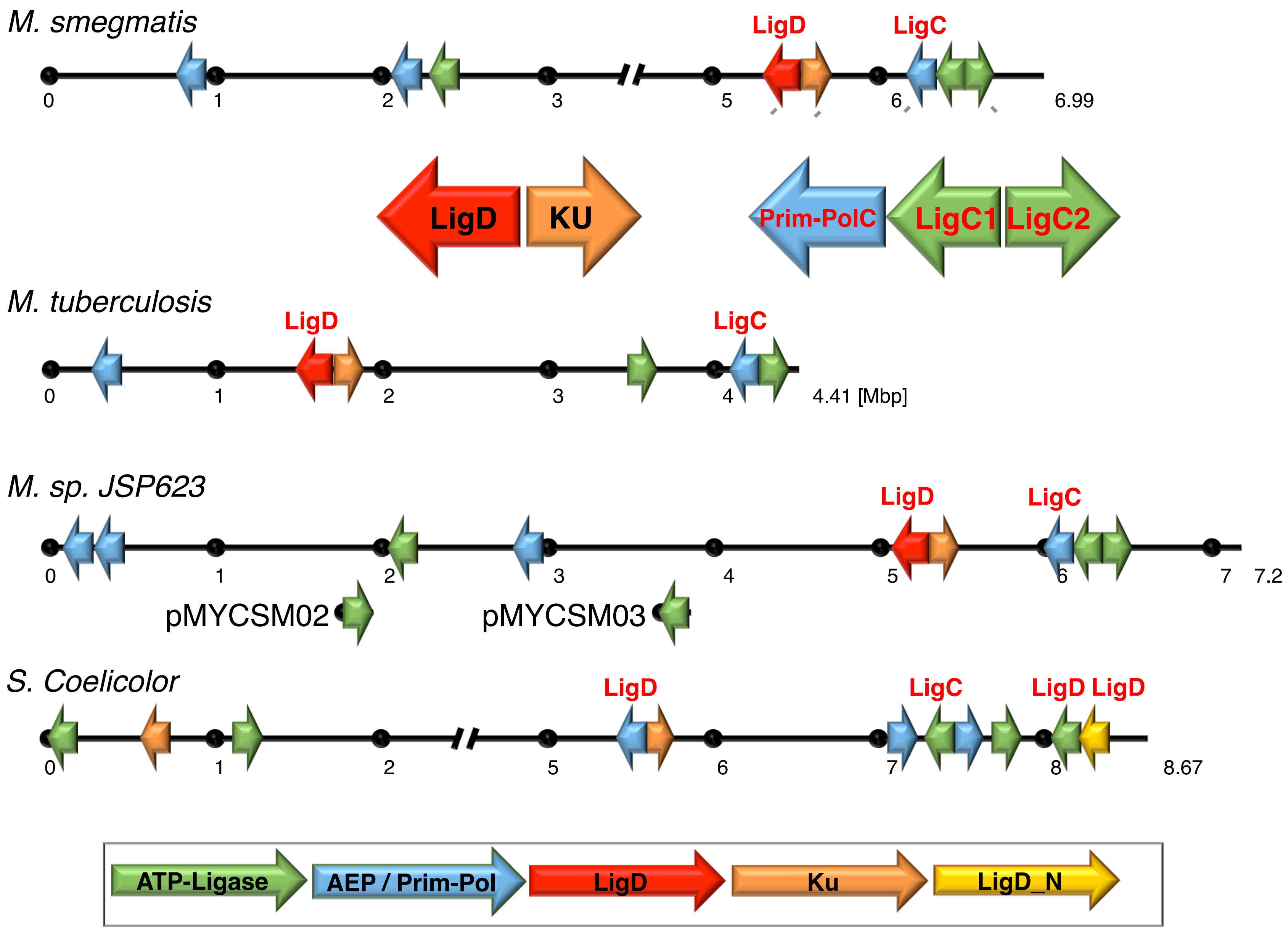
M. smegmatis
LigD
LigC
0
1
2
3
5
6
6.99
LigD
KU
Prim-PolC
LigC1
LigC2
M. tuberculosis
LigD
LigC
0
1
2
3
4
4.41 [Mbp]
M. sp. JSP623
LigD
LigC
0
1
2
3
4
5
6
7
7.2
pMYCSM02
pMYCSM03
S. Coelicolor
LigD
LigC
LigD LigD
0
1
2
5
6
7
8
8.67
ATP-Ligase
AEP / Prim-Pol
LigD
Ku
LigD_N

Supplementary Figure 1. Diversity and genomic localization of Prim-Pol and ATP-dependent DNA ligase genes in *Actinobacteria*. Approximate distribution of genes (depicted as arrows) encoding Prim-Pols (blue), ATP-dependent DNA ligases (green) and non-homologous end joining elements: multidomain LigD (red), Ku protein (orange) and LigD associated phosphoesterase (yellow); are shown on respective actinobacterial genomes and mobile elements (black nodes). The three separate components of the LigD complex in Streptomyces are denoted.

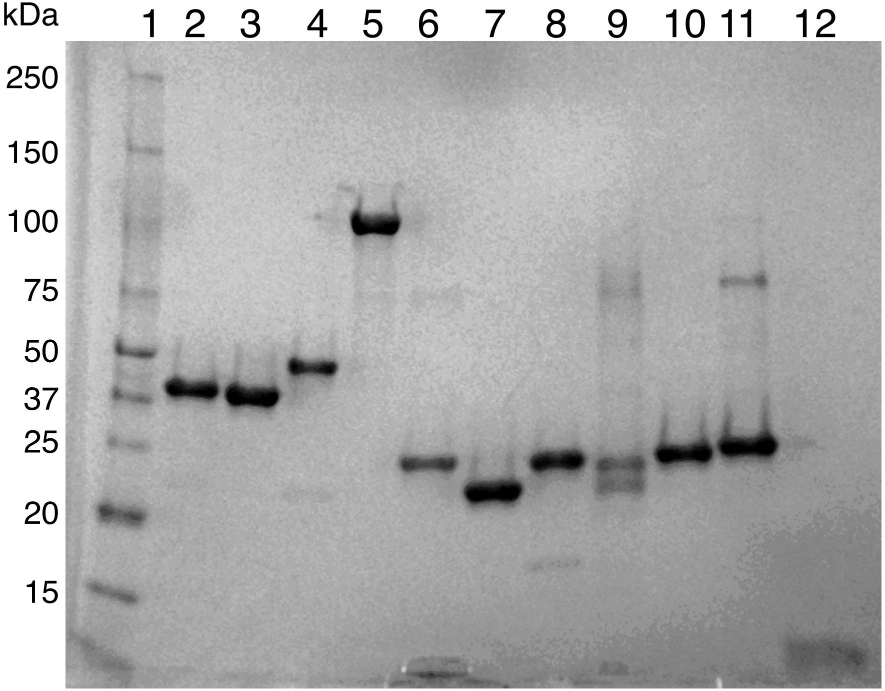
kDa
1 2 3 4 5 6 7 8 9 10 11 12
250
150
100
75
50
37
25
20
15

Supplementary Figure 2. Recombinant proteins purified for this study. Approximately 2μg of each purified recombinant protein was loaded on a 4-12% gradient SDS-polyacrylamide gel and resolved in 1 x TGS buffer under routine conditions. Gel was stained with colloidal Coomassie stain. Proteins loaded as follows: 1. Protein molecular weight marker, 2. LigC1 39,5kDa +HIS, 3. PrimPolC 39,4kDa +HIS, 4. PolD1 47,6kDa +HIS, 5. PolA 99,9kDa +HIS, 6. FPG 31,6kDa +C-HIS, 7. MPG 21,5kDa +HIS, 8. Nth 28,8kDa +HIS, 9. Endo4 26,6kDa +HIS, 10. XthA (MSMEG_0829) 29,1kDa +HIS, 11. ExoIII (MSMEG_1656) 30,1kDa +HIS, 12. XseB 7,8kDa.

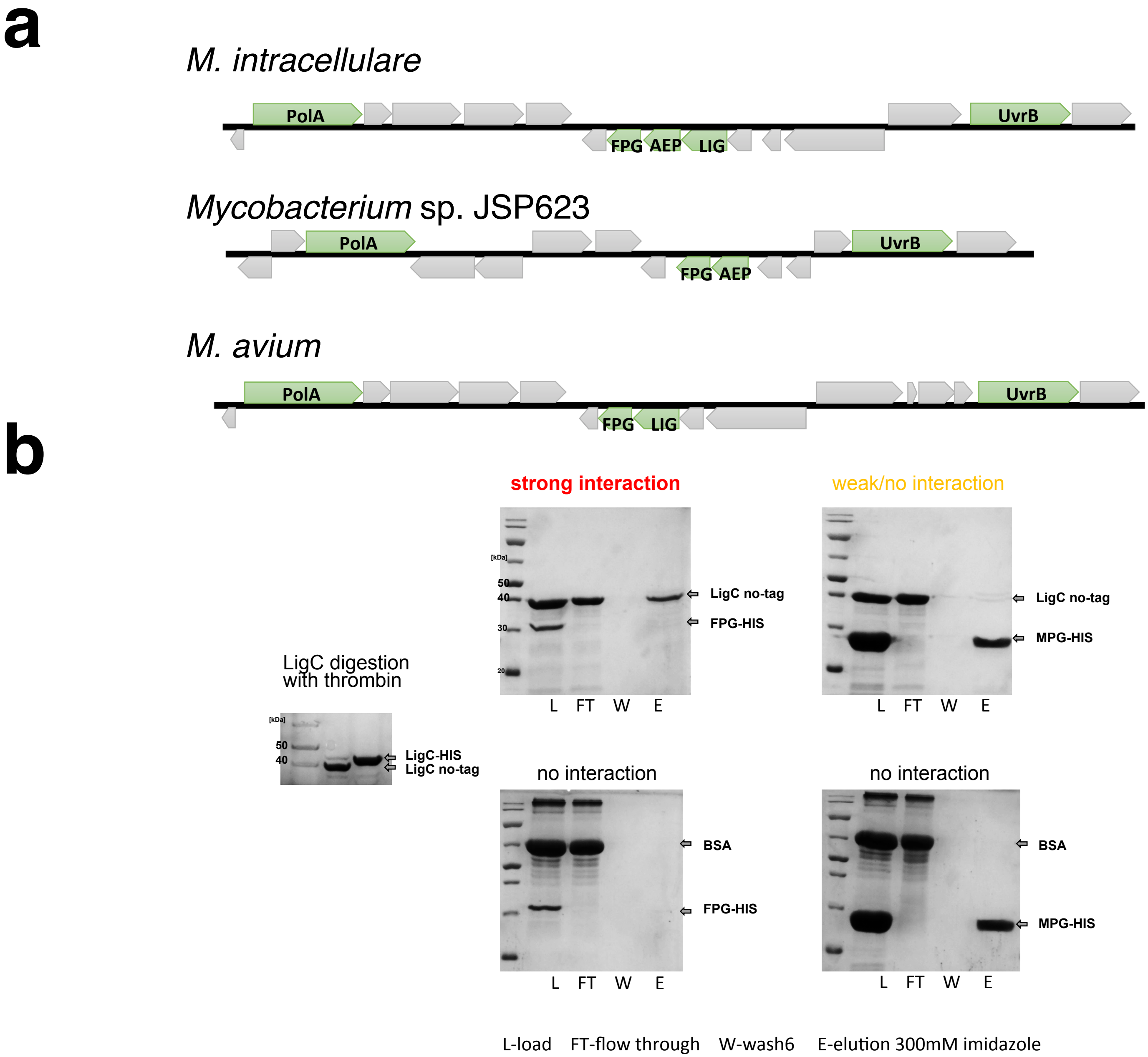
a
M. intracellulare
PolA
UvrB
FPG
AEP
LIG
Mycobacterium sp. JSP623
PolA
UvrB
FPG
AEP
M. avium
PolA
UvrB
FPG
LIG
b
strong interaction
weak/no interaction
[kDa]
50
40
30
20
LigC no-tag
FPG-HIS
LigC no-tag
MPG-HIS
L FT W E
L FT W E
LigC digestion with thrombin
[kDa]
50
40
LigC-HIS
LigC no-tag
no interaction
no interaction
BSA
FPG-HIS
BSA
MPG-HIS
L FT W E
L FT W E
L-load FT-flow through W-wash6 E-elution 300mM imidazole

Supplementary Figure 3. Operonic and functional association of bifunctional glycosylase FPG with Prim-PolC and LigC homologues in mycobacteria.

(a) For chosen mycobacteria, genomic regions encoding FPG co-transcribed with either individual or both Prim-PolC and LigC orthologues, in the vicinity of base excision repair genes *polA* and *uvrB,* are presented. **(b)** Pull-down assay confirmation of the stable protein-protein interaction between recombinant tag-free LigC (prey protein) and FPG-6xHis (bait protein) from *M. smegmatis*, with respective controls.

a

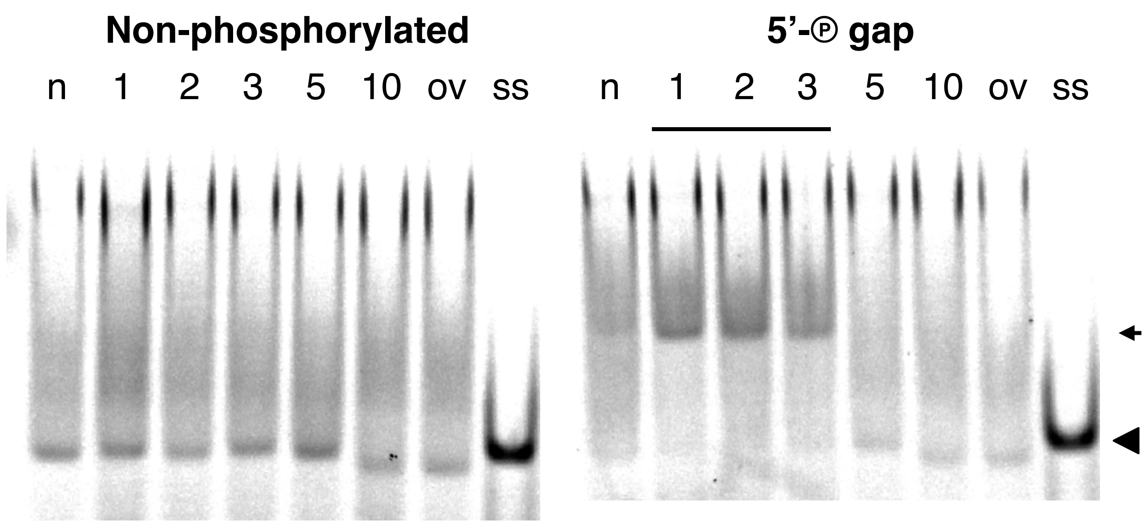
Non-phosphorylated
n 1 2 3 5 10 ov ss
5’-Ⓟ gap
n 1 2 3 5 10 ov ss

b

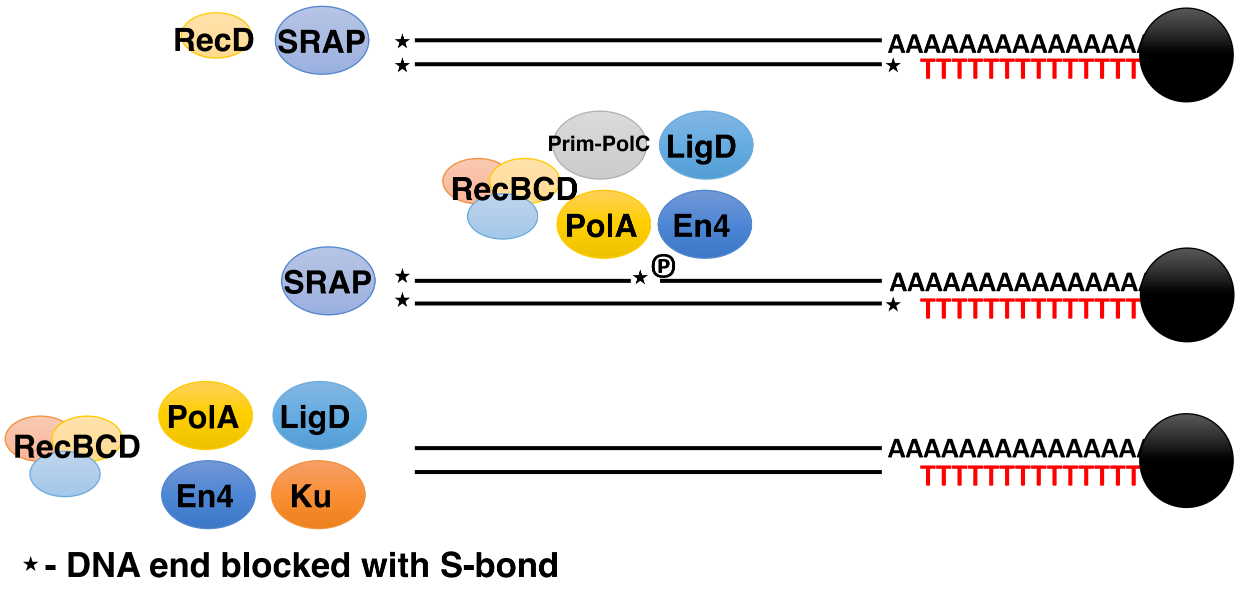
RecD
SRAP
AAAAAAAAAAAAAA
TTTTTTTTTTTTTT
Prim-PolC
LigD
RecBCD
PolA
En4
SRAP
Ⓟ
AAAAAAAAAAAAAA
TTTTTTTTTTTTTT
RecBCD
PolA
LigD
En4
Ku
AAAAAAAAAAAAAA
TTTTTTTTTTTTTT
★ - DNA end blocked with S-bond

Supplementary Figure 4. Substrate specificities of Prim-PolC and LigC1. **(a)** An EMSA showing the optimal substrate binding specificity of Prim-PolC. Reaction mixtures composed of 30nM of substrate, with a nick or single stranded gap of various lengths, with or without phosphorylation of the 5' end of the lesion and 300nM Prim-PolC were incubated on ice for 20 minutes and resolved on a 5% native polyacrylamide gel. A filled triangle indicates the unshifted probe, whereas the arrow indicates a discreet bound complex of PrimPolC and DNA. **(b)** A schematic representation of proteins isolated from mycobacterial extracts that bound to a variety of different DNA substrates coated onto magnetic beads (black filled circles). Proteins were isolated by pulling-down these beads, washing them extensively and subsequently identified by mass spectrometry analysis.

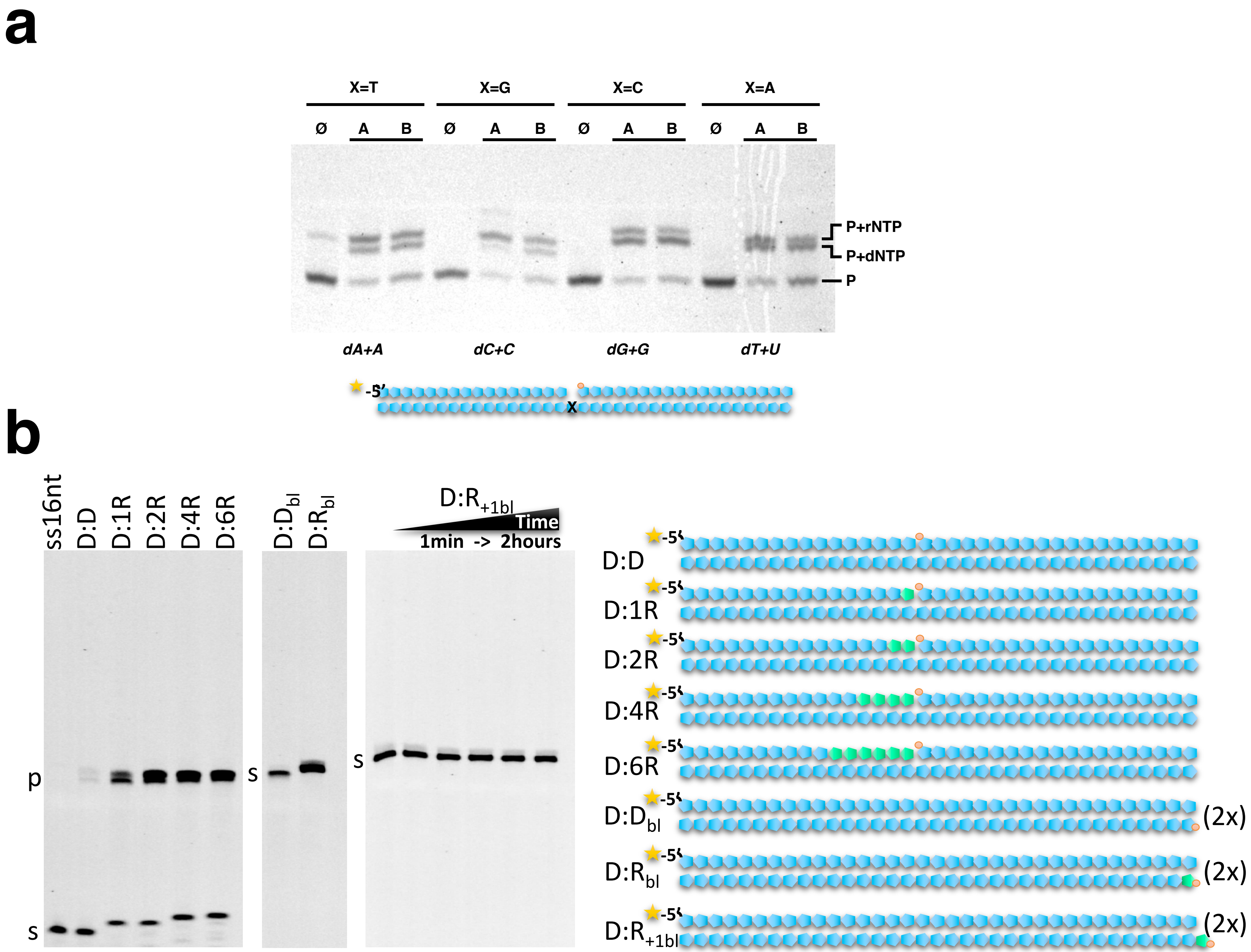

a
X=T
X=G
X=C
X=A
Ø
A
B
P+rNTP
P+dNTP
P
dA+A
dC+C
dG+G
dT+U
-5'
X
b
ss16nt
D:D
D:1R
D:2R
D:4R
D:6R
D:D$_{bl}$
D:R$_{bl}$
D:R$_{+1bl}$
Time
1min -> 2hours
p
s
(2x)

Supplementary Figure 5.

(a) Competition assay for deoxy- versus ribo- nucleotides. The ratio of deoxy : ribo is 100:1 with 50nM : 500pM in A and 5nM : 50pM in B. The schematic of the substrate is depicted below the gel. The templating base is defined with an X and the complementary deoxy : ribo mix is used as the cofactor in a gap-filling reaction. The activity was assayed as described in the methods with the following exceptions; 30°C incubation for 30 minutes with the products resolved on a 25% denaturing gel. The preference factor, F, is given by F = P + rNMP / P + dNMP as determined by densitometry. F was determined for a minimum of 5 experiments at two concentrations and averaged for the four base pairings. **(b)** LigC1 ligation assays. Ligation reactions were carried out in the Tris-pH 7.5 based buffer containing 300nM of LigC1, 0.1mg/ml BSA, 1mM DTT with addition of 1mM ATP, 100μM manganese and 5mM magnesium as cofactors for 45 min at 37°C or up to 2 hours for LigD substrate (D:R_{+1bl}). Substrates used for ligation are depicted as a cartoon on the right side of panel **b**. Phosphorylation of DNA ends is marked with a red circle and green and blue diamonds represent ribo- and deoxyribonucleotides, respectively.

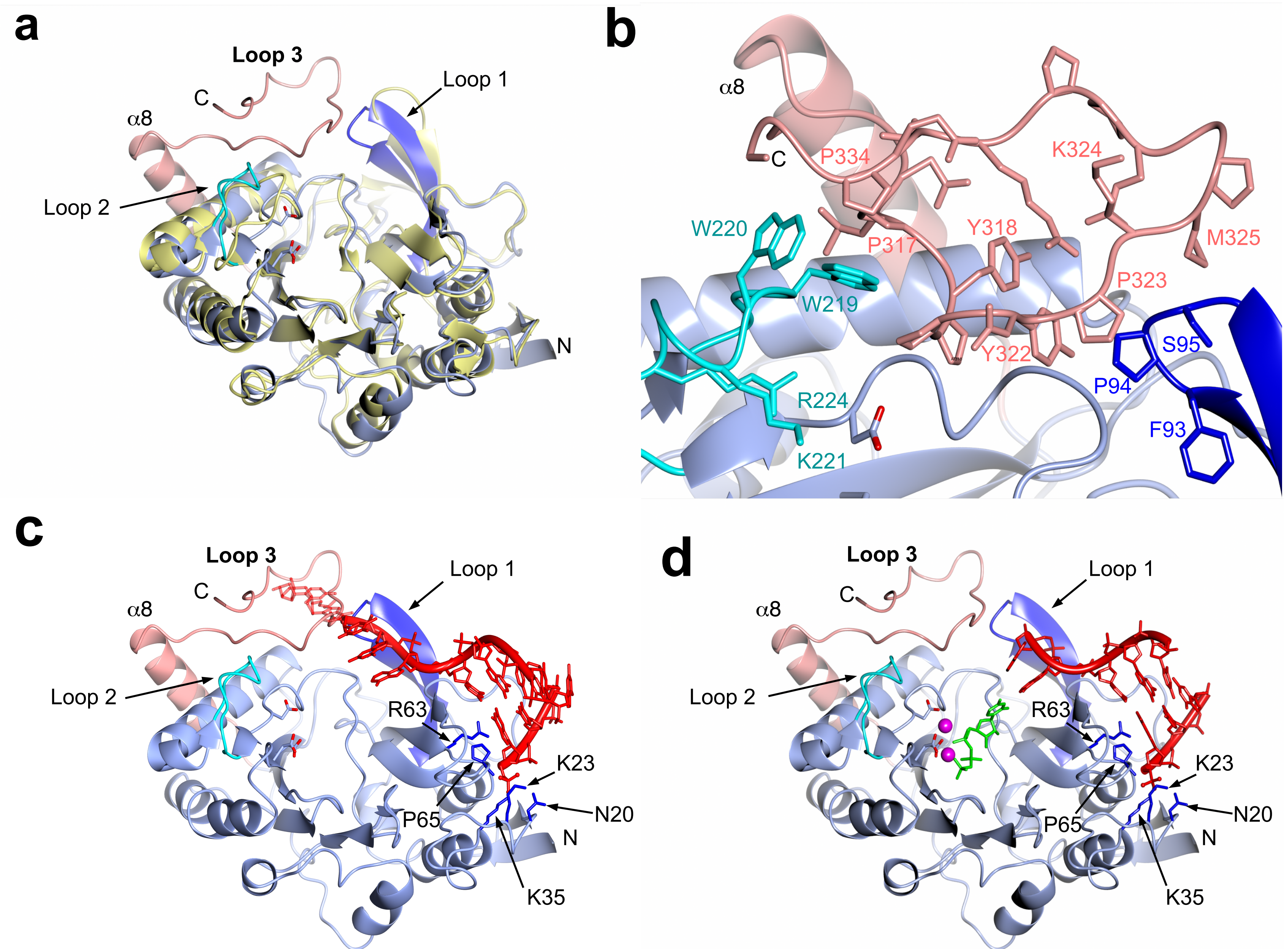
a
Loop 3
C
α8
Loop 1
Loop 2
N
b
α8
C
P334
K324
W220
Y318
P317
M325
P323
W219
S95
Y322
P94
R224
F93
K221
c
Loop 3
Loop 1
C
α8
Loop 2
R63
K23
P65
N20
N
K35
d
Loop 3
Loop 1
C
α8
Loop 2
R63
K23
P65
N20
N
K35

Supplementary Figure S6. Structural differences between Prim-PolC and Prim-PolD. (a) Superposition of *Msm* Prim-PolC (sky blue) and *Mtu* Prim-PolD (lemon, PDBID: 3PKY). Loop 1 and Loop 2 are shown in dark blue and cyan, respectively. The loops occupy differing positions, reinforcing the idea that the functions of the conserved structural elements have developed to stabilize the Loop 3 element (pink). **(b)** Loop 1 and Loop2 residues involved in stabilising Loop 3 are highlighted along with the conserved residues of Loop 2. **(c)** Superposed DNA as in Figure 5D. In this instance, the full path of the template DNA is shown with the DNA (red) that clashes with Loop 3 in translucent form. The conserved residues involved in phosphate binding and DNA interaction are highlighted. **(d)** As C, but the superposed DNA (red), UTP (green) and catalytic metal ions (magenta) are from the *Mtu* Prim-PolD pre-ternary structure (PDBID: 3PKY). This representation illustrates the catalytic core is structurally conserved.

A

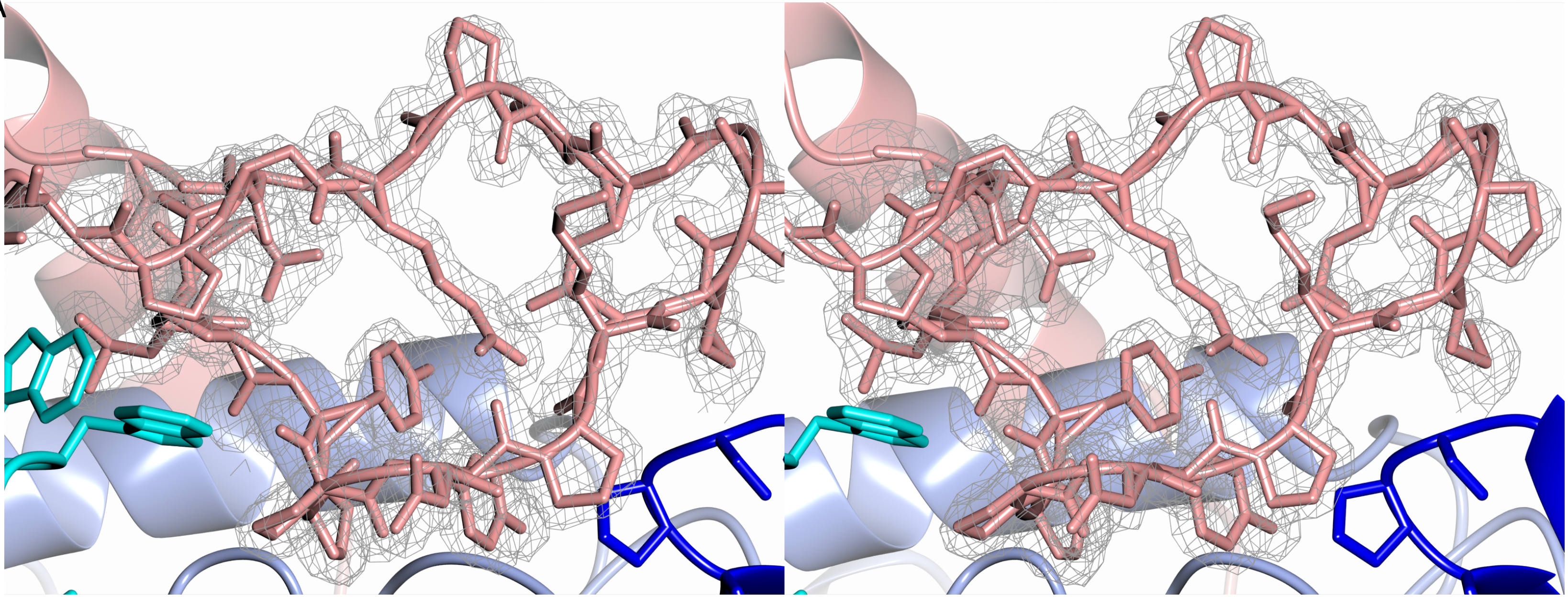

Supplementary Figure S7. Stereo view of electron density for Loop 3 of Prim-PolC. A stick representation of the C-terminal residues G313-P333 that make up Loop 3 (pink). Density from a weighted 2Fo-Fc map scaled at 1.1σ is also depicted in grey. Loop 1 and Loop 2 residues are coloured blue and cyan, respectively.

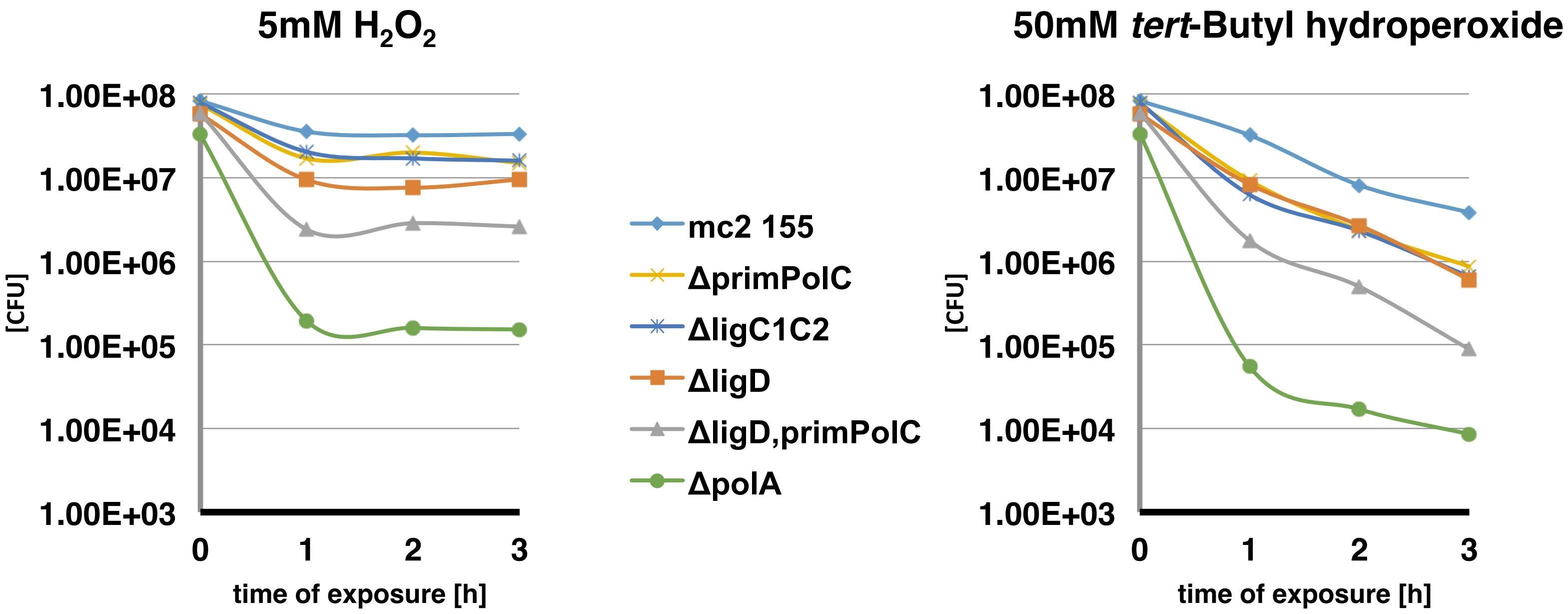
5mM H_2O_2
50mM *tert*-Butyl hydroperoxide
1.00E+08
1.00E+07
1.00E+06
1.00E+05
1.00E+04
1.00E+03
[CFU]
0
1
2
3
time of exposure [h]
mc2 155
ΔprimPolC
ΔligC1C2
ΔligD
ΔligD,primPolC
ΔpolA

Supplementary Figure 8.

Plots of CFU data for phenotypes of mutants lacking Prim-Pols and ATP-dependent DNA ligases treated with inorganic and organic hydroperoxides: 5mM H_2O_2 – hydrogen peroxide and 50mM *tert*-butyl hydroperoxide. Cells were plated at 1, 15, 30 or 60 min after treatment.

Supplementary Figure 9.

Uncropped versions of the western blots and gels used in this study are shown in the panels below.

Figure 1b

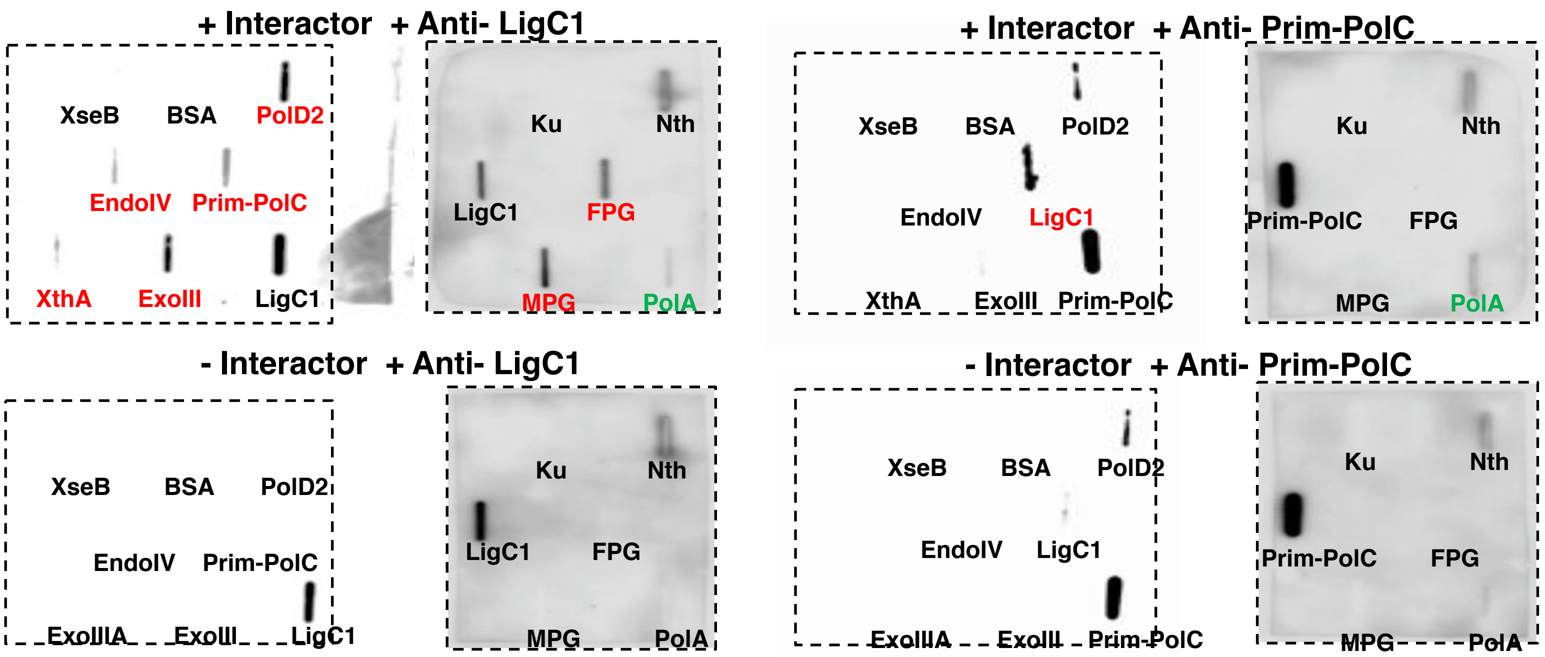

Figure 2a

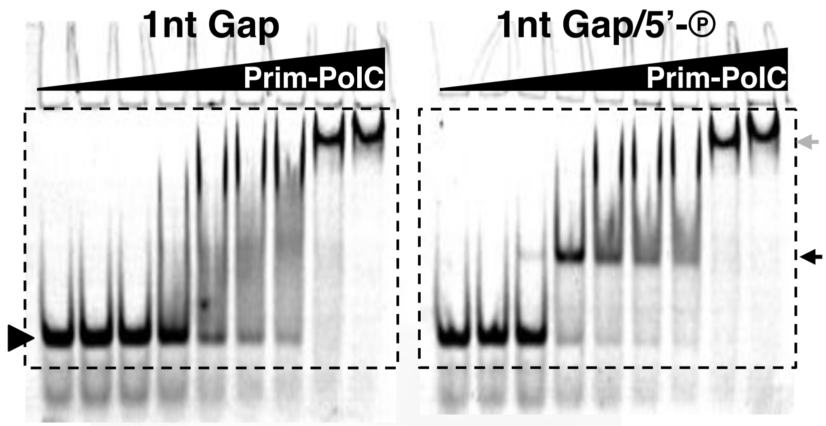

Figure 2b

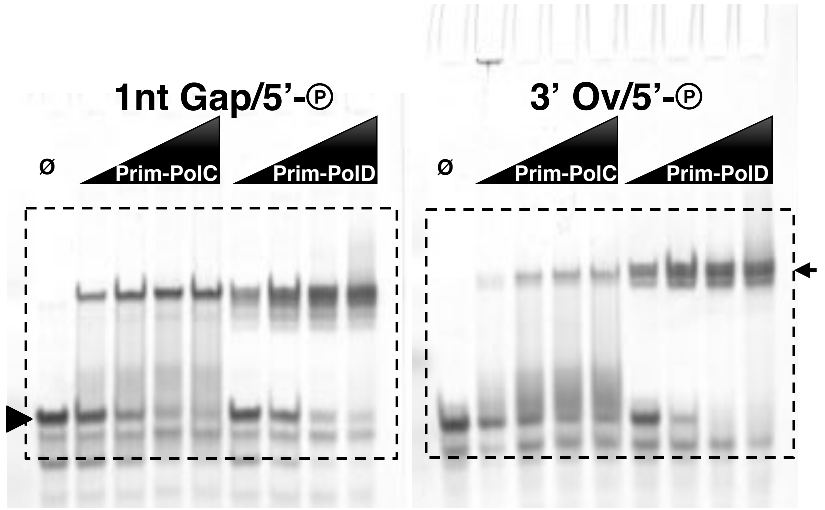

Figure 2c

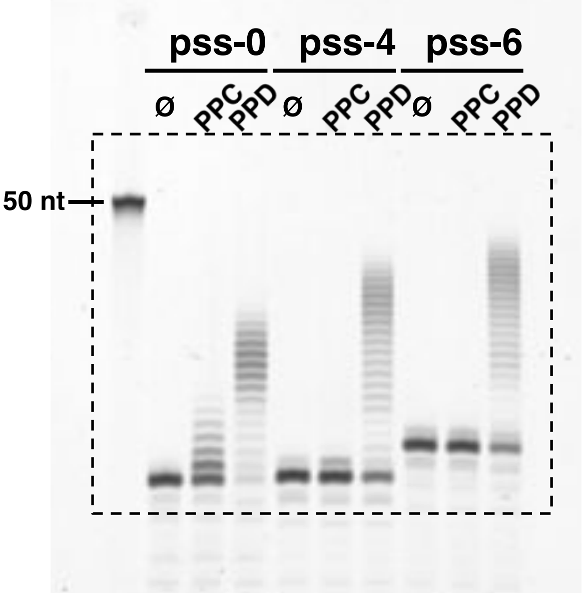

Figure 2d

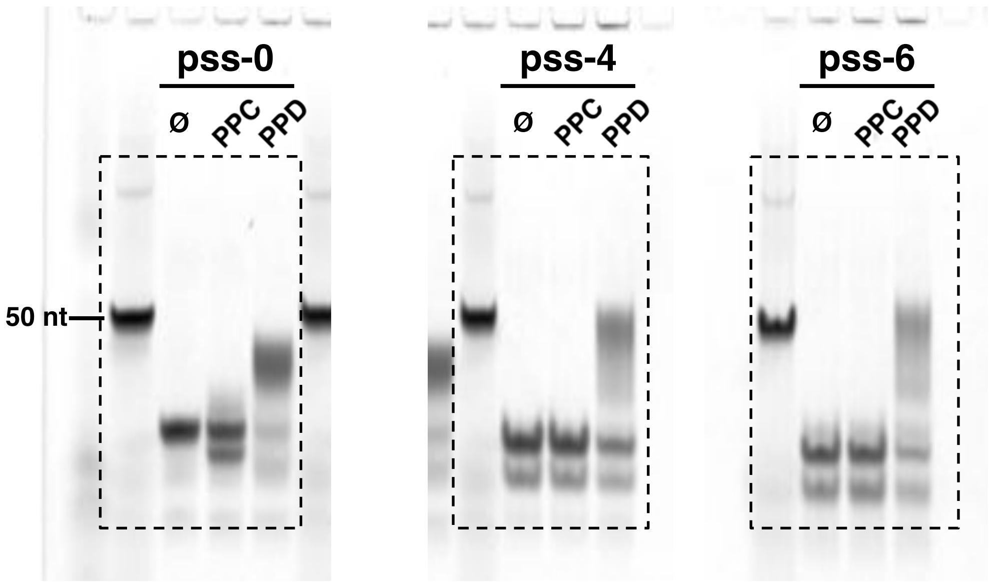

Figure 3a

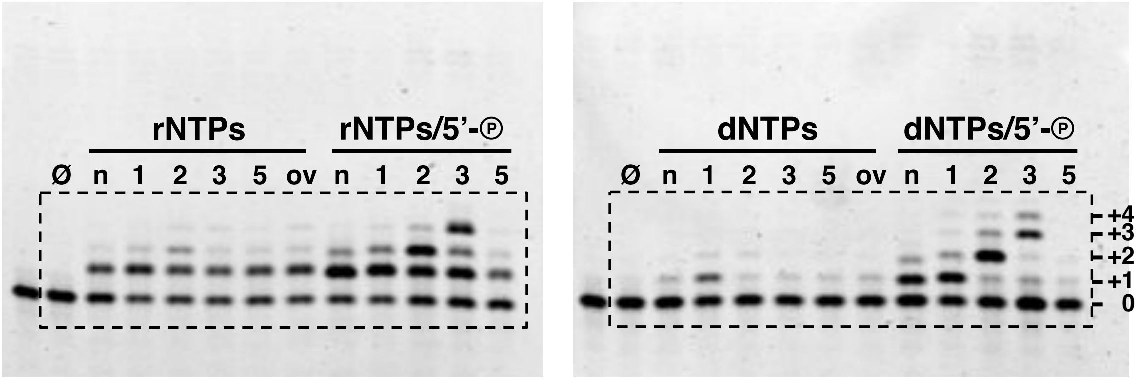

Figure 3b

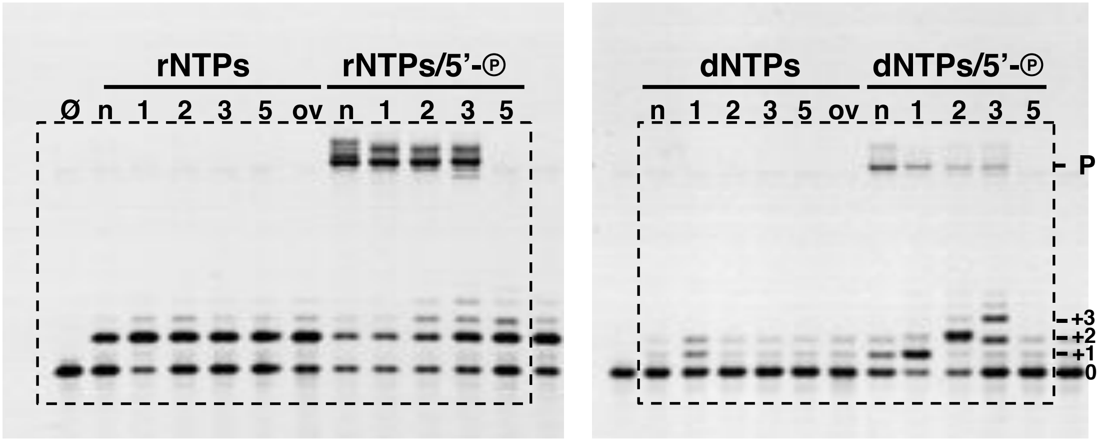

Figure 4b

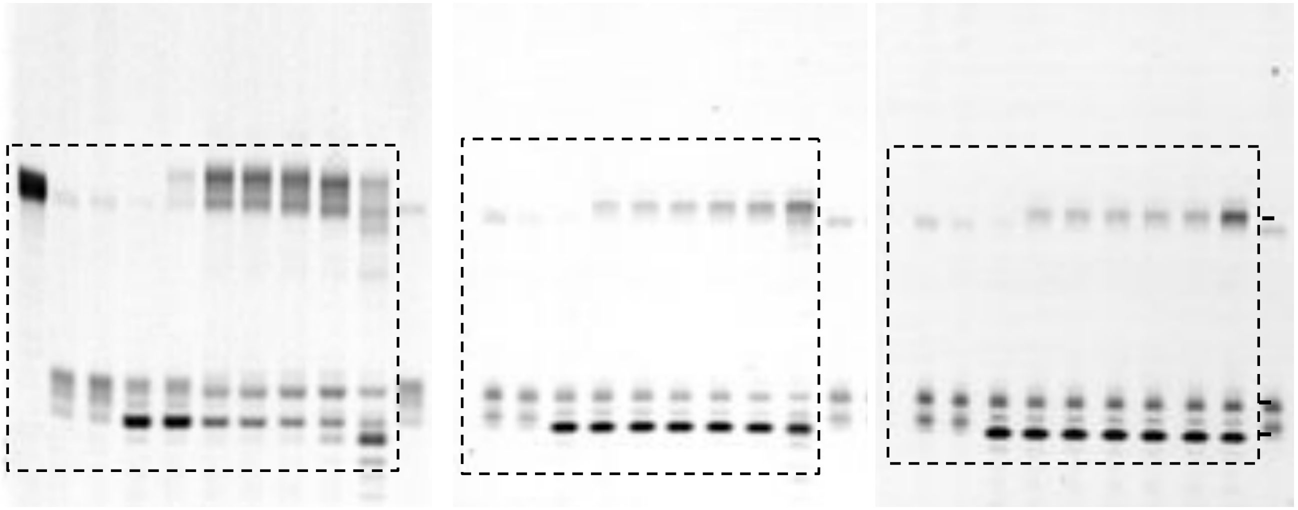

Figure 4c

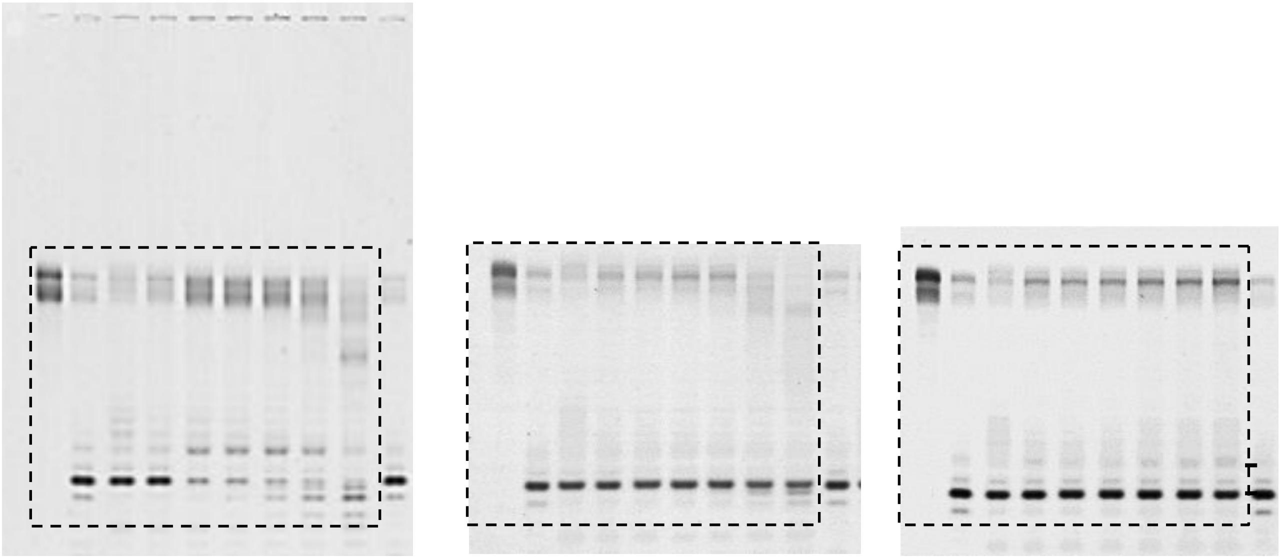

Figure 6a

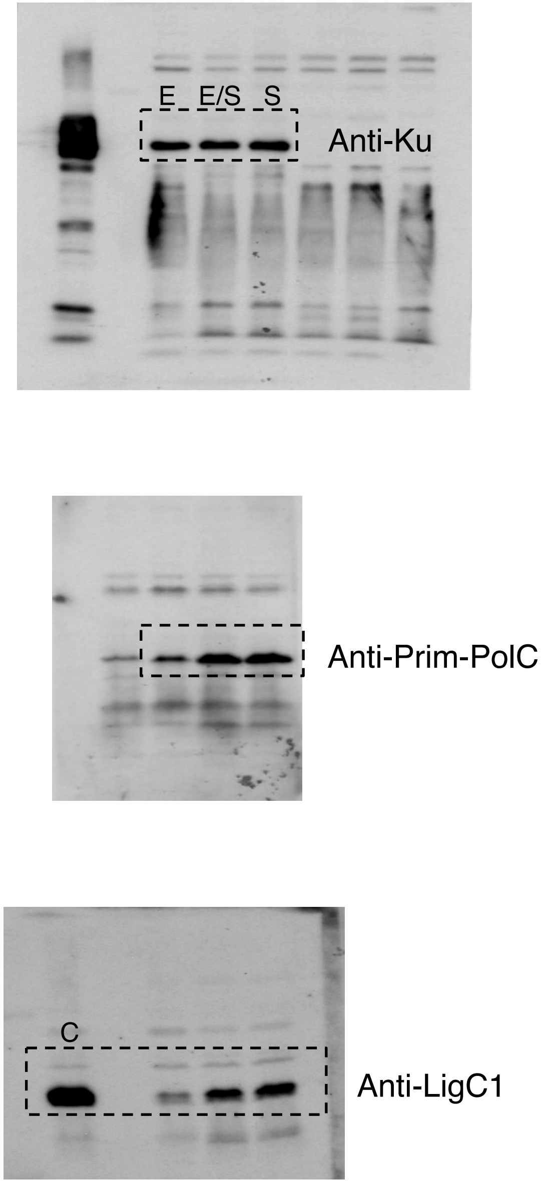

Supplementary Figure 2

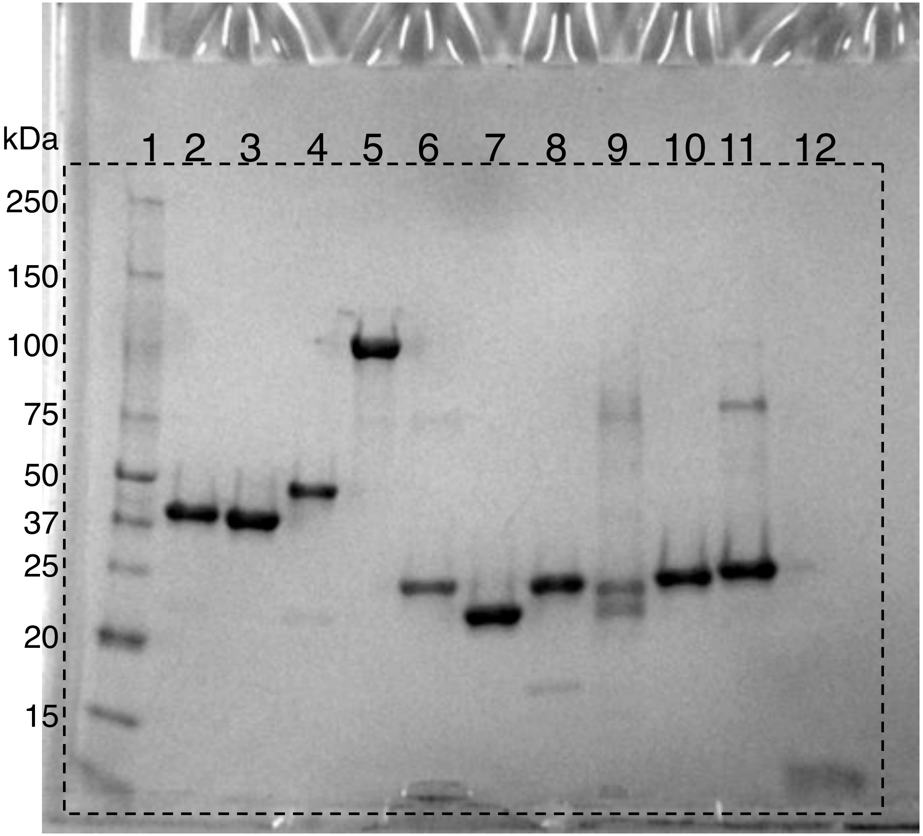

Supplementary Figure 3b

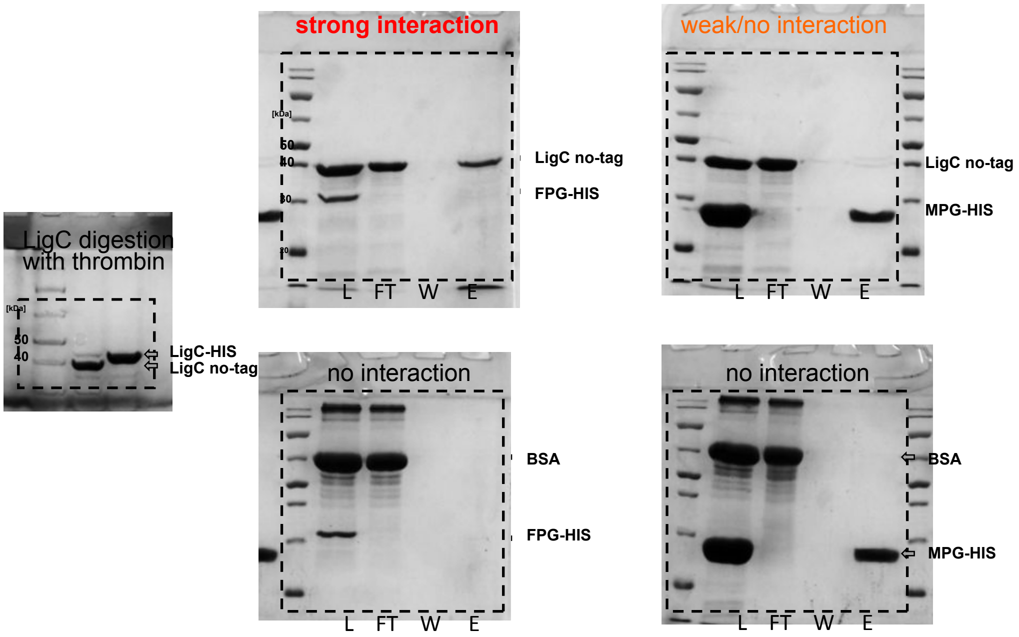

L-load FT-flow through W-wash6 E-elution 300mM imidazole

Supplementary Figure 4a

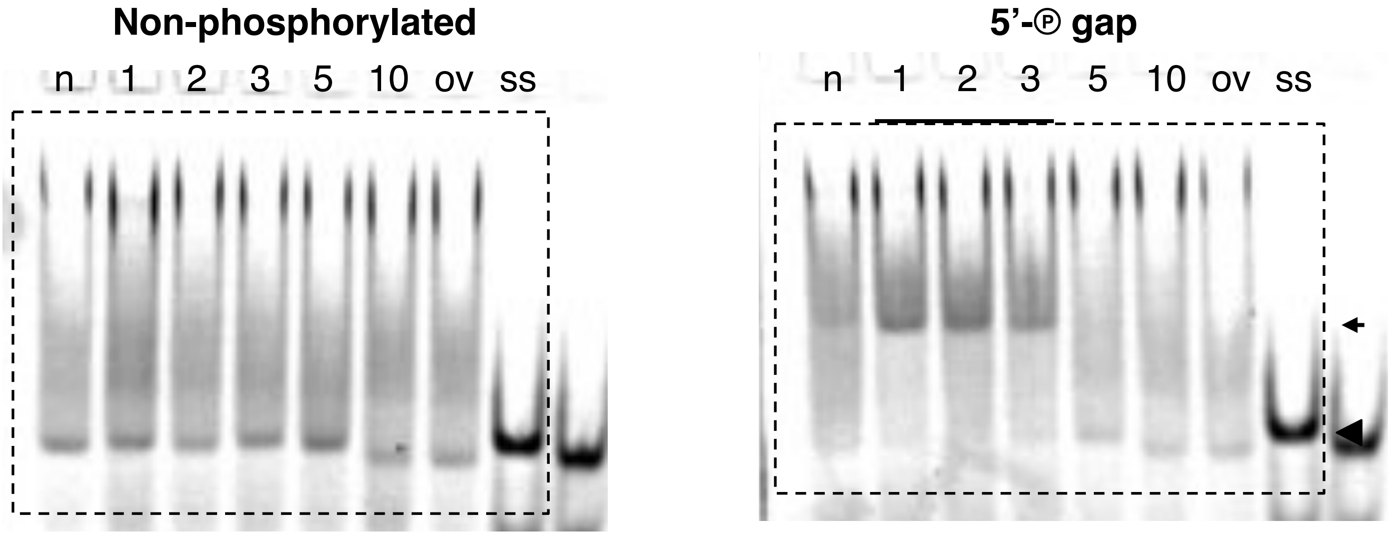

Supplementary Figure 5a

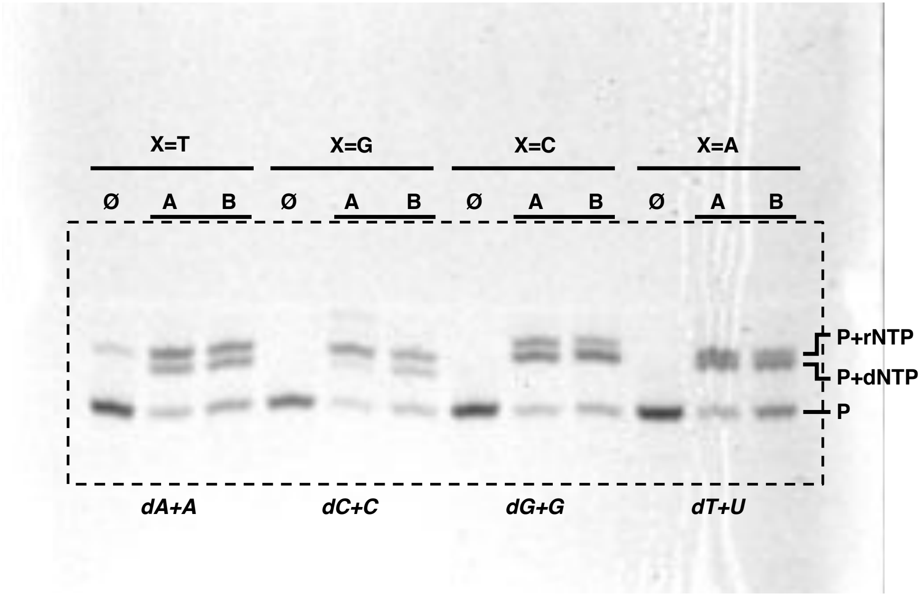

Supplementary Figure 5b

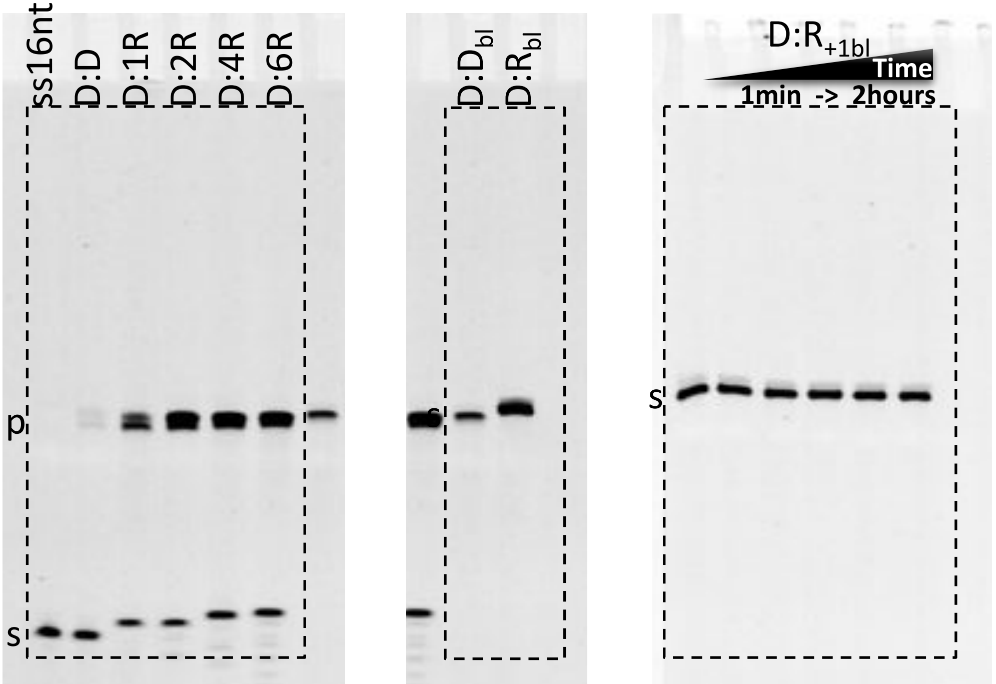

Supplement: Supplementary file 1 — Supplementary Information [file 41467_2017_1365_MOESM1_ESM.pdf]
