# Supplementary material for: DNA Ligase C and Prim-PolC participate in base excision repair in mycobacteria
Source: Nat Commun. 2017 Nov 1;8:1251. doi: 10.1038/s41467-017-01365-y (PMC5663958; doi:10.1038/s41467-017-01365-y)
Supplement: Supplementary file 3 — Description of Additional Supplementary Files [file 41467_2017_1365_MOESM3_ESM.pdf]

Description of Additional Supplementary Files

File Name: Supplementary Data 1

Description: Full list of preys co-purified in eGFP facilitated purifications of protein complexes formed in vivo by Prim-PolC and LigC1 used as baits.

File Name: Supplementary Data 2

Description: Mascot list of preys captured using mycobacterial eGFP-tagged endonuclease IV as bait in M. bovis BCG. Results show copurification of LigC, confirming that these two proteins form a complex in vivo.

File Name: Supplementary Data 3

Description: Protein identification details for AP-MS experiments of preys co-purified on a substrate containing a single nucleotide gap. MaxQuant derived label free protein intensities are provided.
